# The effect of COVID-19 on placental functioning in South African pregnancies: investigation of kisspeptin expression and vascular and inflammatory alterations

**DOI:** 10.1007/s00418-025-02381-6

**Published:** 2025-05-05

**Authors:** C. Heeralall, U. H. Ibrahim, M. Jenneker, S. Singh, M. Matjila, L. Lazarus, I. Mackraj

**Affiliations:** 1 Discipline of Clinical Anatomy, School of Laboratory Medicine and Medical Sciences, College of Health Sciences, University of KwaZulu-Natal, Durban, South Africa; 2 Discipline of Human Physiology, School of Laboratory Medicine and Medical Sciences, College of Health Sciences, University of KwaZulu-Natal, Durban, South Africa; 3 Discipline of Obstetrics and Gynecology, School of Clinical Medicine, University of KwaZulu-Natal, Durban, South Africa; 4 Department of Obstetrics and Gynecology, Faculty of Health Sciences, University of Cape Town, Groote Schuur Hospital, Cape Town, 7925 South Africa

**Keywords:** COVID-19, Placenta, Kisspeptin, Implantation, Inflammation

## Abstract

The coronavirus disease 2019 (COVID-19) pandemic has passed; however, its long-term effects are yet to be determined. Pregnant women and their neonates faced a higher risk for complications during this pandemic as COVID-19 was reported to result in oxidative and inflammatory stress and the cytokine storm, which would impact pregnancy, namely the trophoblast invasion and placental development and functioning. Therefore, this study aims to determine the effect of COVID-19 on the placental functioning in South African pregnancies through the analysis of kisspeptin and placental morphology. Immunohistochemical analyses of placental samples were performed to detect the expression of kisspeptin. Histopathological analysis was conducted to identify vascular and inflammatory alterations. This study demonstrated that COVID-19 results in a significantly increased expression of placental kisspeptin in both the central (*p* = 0.001) and peripheral (*p* < 0.0001) regions as compared with the placentae from control pregnancies. Upon further analysis, the placentae from COVID-19 pregnancies also presented with severe inflammation and maternal and fetal vascular malperfusion compared with the control placentae. A significantly increased expression of placental kisspeptin was observed in COVID-19 positive pregnancies, implying impaired placental functioning. This was further supported by vascular and inflammatory alterations observed in COVID-19-positive placentae, which may suggest that trophoblast invasion was compromised. To date, there still exists small clusters of COVID-19 outbreaks, and our findings highlight the importance of the future surveillance of these mothers and neonates in COVID-19 pregnancies in South Africa, as neonates from other countries have presented with abnormalities.

## Introduction

The coronavirus disease 2019 (COVID-19) pandemic, caused by severe acute respiratory syndrome coronavirus 2 (SARS-CoV-2), has led to millions of confirmed cases globally, with South African recording the highest prevalence on the African continent (WHO 2024). The outbreak of SARS-CoV-2 emerged in late 2019 and resulted in a widespread pandemic, which continues to have significant health, social, and economic consequences (Chuang et al. 2024; Gheorghita et al. 2024). In the USA, over 400,000 women have been impacted by this pandemic, including 23,434 pregnant women (Zambrano et al. 2020). Given the ongoing challenges posed by COVID-19, it remains crucial to investigate the effects of this virus on pregnant women and fetal health, as long-term consequences on maternal and fetal health are not yet fully understood.

The severity of COVID-19 ranged from fevers and fatigue to pneumonia with decreased oxygen saturation (Velavan and Meyer 2020). These symptoms were further exacerbated in pregnant women, as compared with nonpregnant women, who were at higher risk of being admitted into intensive care (Wenling et al. 2020; Zambrano et al. 2020). These women were reported to be more vulnerable to pneumonia and other respiratory diseases owing to differences in oxygen consumption and T lymphocyte immunity compared with that of a normal individual (Wenling et al. 2020; Zambrano et al. 2020). In addition, COVID-19 could further induce acute inflammation in pregnant mothers, causing respiratory distress and organ failure that could impact fetal development (Granja et al. 2021; Seymen 2021). Moreover, adverse consequences in the placenta of these pregnant women as a result of COVID-19 were observed, which presented as signs of inflammation, vascular alterations, hypoxia with an increased risk of preeclampsia (PE), preterm birth, and stillbirth (Wenling et al. 2020; Abedzadeh‐Kalahroudi et al. 2021; Jamieson and Rasmussen 2021; Wastnedge et al. 2021; Khoiwal et al. 2022).

Proper implantation and placental development and functioning are fundamental for the success of all pregnancies; thus, understanding if these processes were impacted in COVID-19 pregnancies would be beneficial to understand the outcomes and long-term impact associated with these pregnancies (Boss et al. 2018). Any alteration from as early as implantation has the potential to result in abnormal placentation (Burton and Jauniaux 2023). There is an increase in evidence suggesting that, as a result, complications can extend beyond gestation as it has been further linked to predisposing infants to neuropsychiatric, metabolic, and cardiovascular disorders (Burton and Jauniaux 2023). Trophoblast migration and invasion are fundamental to implantation, required for the development of the placenta and fetus, and are dependent on factors such as oxygen tension and angiogenic and growth factors (Matjila 2015; Silva and Serakides 2016; Kapustin et al. 2020). Kisspeptins (a group of peptide fragments encoded by the metastasis suppressor gene, namely, *KISS-1*) are key components in trophoblast migration and invasion that possess a great influence on placental formation and development (Hu et al. 2019; Akhtar et al. 2020) through an array of mechanisms, including the inhibition of certain matrix metalloproteinase (MMP) enzymes (Cao et al. 2019; Gorbunova and Shirshev 2020). The invasion of extravillous trophoblasts into the maternal tissue is a pivotal factor in the oxygenation of the placenta and fetus to prevent hypoxia (Huppertz et al. 2014). Alterations in kisspeptin expression have been linked to PE, preterm birth, intrauterine growth restriction (IUGR), and an increased risk for miscarriage (Cao et al. 2019; Kapustin et al. 2020). Kisspeptin has been shown to significantly influence preeclampsia, in which insufficient invasion can result in hypoperfusion, the release of proinflammatory markers, and poor vascular remodeling (Gomes and Sones 2021). Therefore, investigations involving kisspeptin in COVID-19 pregnancies would provide an understanding of the effect that COVID-19 infection has on placental formation and functioning. This could possibly be further linked to the multitude of adverse pathological alterations observed in the placentae from COVID-19 pregnancies.

Several studies have reported histopathological alterations in the placentae from COVID-19 pregnancies (Heeralall et al. 2023), including signs of maternal and fetal malperfusion (Algarroba et al. 2020; Baergen and Heller 2020; Hecht et al. 2020; Hosier et al. 2020; Mulvey et al. 2020; Shanes et al. 2020; Sisman et al. 2020; Smithgall et al. 2020; Hsu et al. 2021; Patberg et al. 2021; Schwartz et al. 2021, 2022; Watkins et al. 2021; Huynh et al. 2022; Mao et al. 2022). Similarly, some studies documented inflammatory changes in the placenta (Baud et al. 2020; Facchetti et al. 2020; Mongula et al. 2020; Pulinx et al. 2020; Richtmann et al. 2020; Vivanti et al. 2020; Gao et al. 2021; Giordano et al. 2021; Ikhtiyarova et al. 2021; Jaiswal et al. 2021; Jang et al. 2021; Liu et al. 2021; Menter et al. 2021; Shchegolev et al. 2021; Dubucs et al. 2022; Kato et al. 2022) with no placental expression studies linking hypoperfusion to kisspeptin. Furthermore, these studies were conducted globally, with limited information available on the placental alterations caused by COVID-19 on the African population, particularly in South Africa (Govender et al. 2021).

Therefore, this study aims to assess the expression of kisspeptin, coupled with morphological changes, in the placentae of COVID-19-positive pregnant South African women. To our knowledge, this is the first study to investigate the effect COVID-19 on kisspeptin levels in pregnant women. These findings have then been further linked to the results from extensive analysis of the morphological structure and alterations in these placentae. The results obtained from the immunohistochemical analysis of kisspeptin together with the histological analysis of the morphology of placentae from COVID-19-positive and COVID-19-negative pregnant women are reported herein.

## Materials and methods

### Ethics approval and consent to participate

Regulatory ethical and institutional approval was obtained from the Biomedical Research Ethics Committee of the University of KwaZulu-Natal (no. BREC/00004591/2022), South Africa. Patients were recruited on the basis of the inclusion and exclusion criteria. Once suitable patients were identified for this study, its purpose and requirements were explained to them. Upon agreeing to participate, all participants signed a consent form.

### Study population (inclusion and exclusion criteria)

The Inkosi Albert Luthuli Central Hospital was under the triage system for COVID-19 diagnoses, and therefore, upon arrival, all patients were tested prior to admission. Black South African women over 34 weeks of gestation who were confirmed to be either COVID-19-positive (*n* = 6 central and *n* = 6 peripheral) or COVID-19-negative (*n* = 6 central and *n* = 6 peripheral) for SARS-CoV-2 RNA via real-time polymerase chain reaction (RT-PCR) on nasopharyngeal swabs were considered for this study. Only those who could provide full consent and did not present with other major comorbidities such as hypertension, preeclampsia, IUGR, or other major health issues were then recruited owing to overlapping pathology.

### Placental tissue processing

Placental samples were collected from women who underwent elective delivery by cesarean section. A central and peripheral piece (including chorionic and basal plate) of placental tissue (*n* = 24) was collected from COVID-19-positive and COVID-19-negative patients. The placental tissue was immediately dissected, rinsed with phosphate-buffered saline (PBS) (1×, pH 7.5), fixed in 10% phosphate-buffered formalin, and embedded into paraffin wax blocks as per standard laboratory procedure (Burton et al. 2014). These samples were then transported to University of KwaZulu-Natal for further processing.

Using a rotary microtome, 3-µm thin sections of placental tissue were floated onto frosted glass slides, deparaffinized, and rehydrated for hematoxylin and eosin (H&E) and Masson’s trichrome (MT) staining according to the methodology described in *Bancroft’s Theory and Practice of Histological Techniques* (Fischer et al. 2008; Suvik and Effendy 2012; Suvarna et al. 2018).

### Immunohistochemistry

The slides were incubated overnight at 37 °C, then dewaxed and rehydrated. Endogenous peroxidase activity was blocked with H_2_O_2_ for 15 min, and the slides were washed with distilled water after. Antigen retrieval was then performed using Tris–ethylenediaminetetraacetic acid (EDTA) buffer (pH 9) for 30 min in a pressure cooker. The slides were then washed in Tris-buffered saline (TBS) and subsequently blocked with normal donkey serum. Incubation with a primary antibody, monoclonal (rabbit immunoglobulin (Ig)G) against kisspeptin ([EPR23770-189], Abcam) at a dilution of 1:500, was conducted at 4 °C overnight. The manufacturer validated the primary antibody using human testis and placenta tissue. COVID-19-negative placental tissue was utilized as method controls and was incubated with and without the primary antibody. Negative controls involved substituting the primary antibody with diluent (normal donkey serum).The next morning, the slides were washed in TBS. Thereafter the secondary antibody, namely, peroxidase-conjugated goat anti-rabbit IgG (1: 100) (Dako catalog no. P0448), was applied. The slides were then washed again with TBS and thereafter incubated for 10 min with 2 drops/1 ml of 3,3′-diaminobenzidine (DAB) substrate chromogen system (DAKO K3468, Agilent, USA) at room temperature. Thereafter, the slides were washed again to counterstain with hematoxylin for 5 min and then rinsed, dehydrated, and cleared with xylene. Mounting medium was then applied to the slides along with a coverslip.

### Morphometric analysis

H&E and MT slides were scanned with the Leica LV1 scanner and captured using Aperio ImageScope 12.4.6 (Leica Biosystems, Germany). Placental sections stained with kisspeptin were viewed with the Axioscope A1 microscope, N-Achroplan 20×, with numerical aperture 0.45 (Carl Zeiss, Germany). A total of four fields of view per slide were selected, and images were captured at 20× objective magnification using AxioVision software (Carl Zeiss, Germany; version 4.8.3). The percentage of immunostaining specific to kisspeptin antibody expression was quantified using color deconvolution on Fiji ImageJ software (Jensen 2013; Crowe and Yue 2019). Color deconvolution involves separating the colors of an image into three channels: red, green, and blue. For this investigation, the red channel represents DAB staining and blue represents hematoxylin staining. The percentage of kisspeptin expression was determined by dividing the percentage of DAB staining by the total tissue area.

### Statistical analysis

Patient demographics were examined for normality using the D’Agostino and Pearson, Shapiro–Wilk, and Kolmogorov–Smirnov tests. To compare the differences between the control and COVID-19-positive groups, the unpaired *t*-test was used. Statistical significance was defined as a probability threshold of *p* = 0.05. GraphPad Prism 8.4.3 (San Diego, CA) was used for all statistical analyses.

## Results

### Clinical characteristics

Relevant clinical information about each group, i.e., control and COVID-19 positive, are summarized in Table 1 as mean ± standard deviation (sd) owing to their parametric distribution. Maternal age, maternal weight, systolic blood pressure, diastolic blood pressure, heart rate, and gestational age were not significantly (ns) different between the control and COVID-19-positive study groups, except for body temperature, which was elevated in the COVID-19-positive group (*p* = 0.460), as shown in Table 1.

**Table 1:** Patient demographics of control and COVID-19-positive pregnant women

	Maternal age (years)	Maternal weight (kg)	Systolic blood pressure (BP) (mmHg)	Diastolic BP (mmHg)	Heart rate (beats per minute)	Body temperature (°C)	Gestational age (weeks)	Birthweight (kg)	Neonatal distress
Control (*n* = 6)	30.67 (± 6.82)	119.7 (± 39.52)	114.2 (± 18.33)	73.17 (± 19.14)	96.50 (± 21.68)	36.17 (± 0.21)	37.17 (± 1.72)	2.93 (± 0.83)	1/6 (16.67%)
COVID-19 positive (*n* = 6)	25.50 (± 4.64)	99.78 (± 36.95)	121.5 (± 14.25)	70.17 (± 9.22)	81.33 (± 14.69)	36.42 (± 0.17)	37.00 (± 1.90)	2.65 (± 0.91)	3/6 (50%)
Significance	ns	ns	ns	ns	ns	*p* = 0.460 *	ns	ns	

Key: results are represented as the mean (± sd), *p < 0.05

### Immunolocalization of placental kisspeptin

**Fig. 1:**
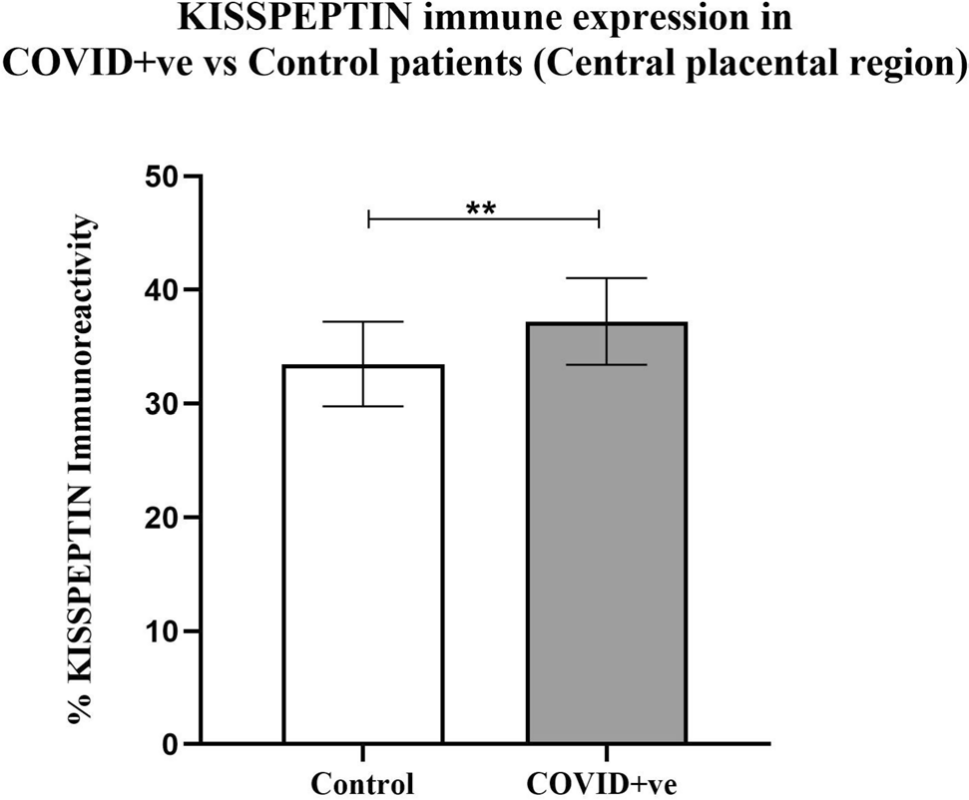
The expression of kisspeptin in the central placental region of COVID-19-positive and negative COVID-19-pregnant women (*n* = 6 per group)

**Fig. 2:**
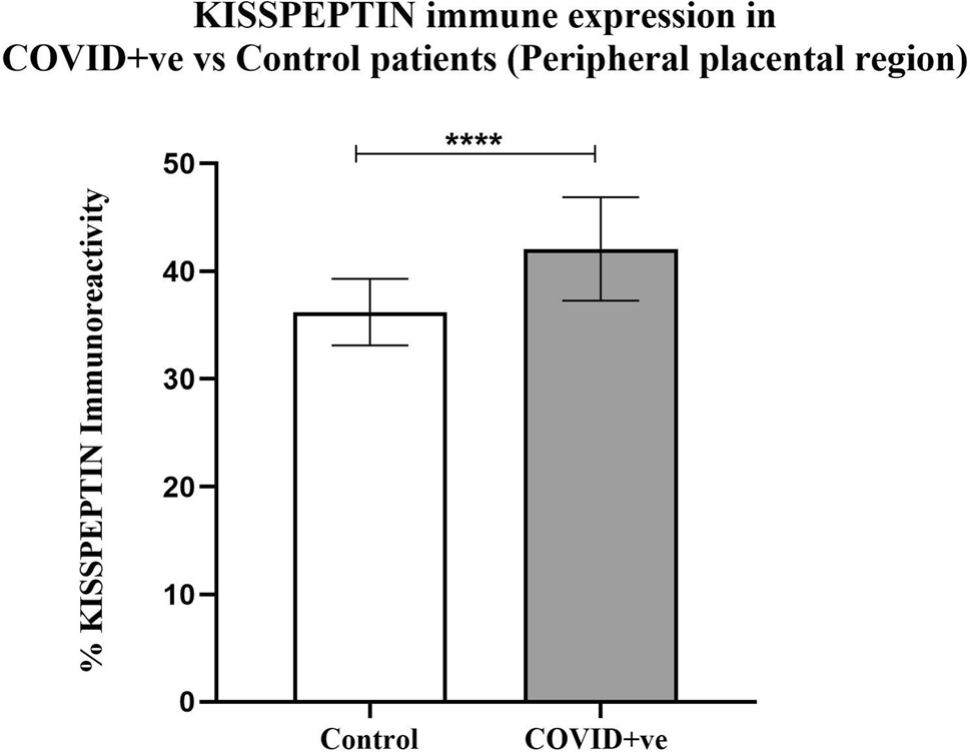
The expression of kisspeptin in the peripheral placental region of COVID-19-positive and COVID-19-negative pregnant women (*n* = 6 per group)

The expression of kisspeptin was localized in the central (Fig. 1) and peripheral (Fig. 2) regions of the placentae obtained from COVID-19-positive and COVID-19-negative pregnant women. The immunoexpression of kisspeptin was significantly increased in both the central (*p* = 0.001) and peripheral (*p* < 0.0001) placental regions from pregnancies with COVID-19 infection as compared with healthy pregnancies.

**Fig. 3:**
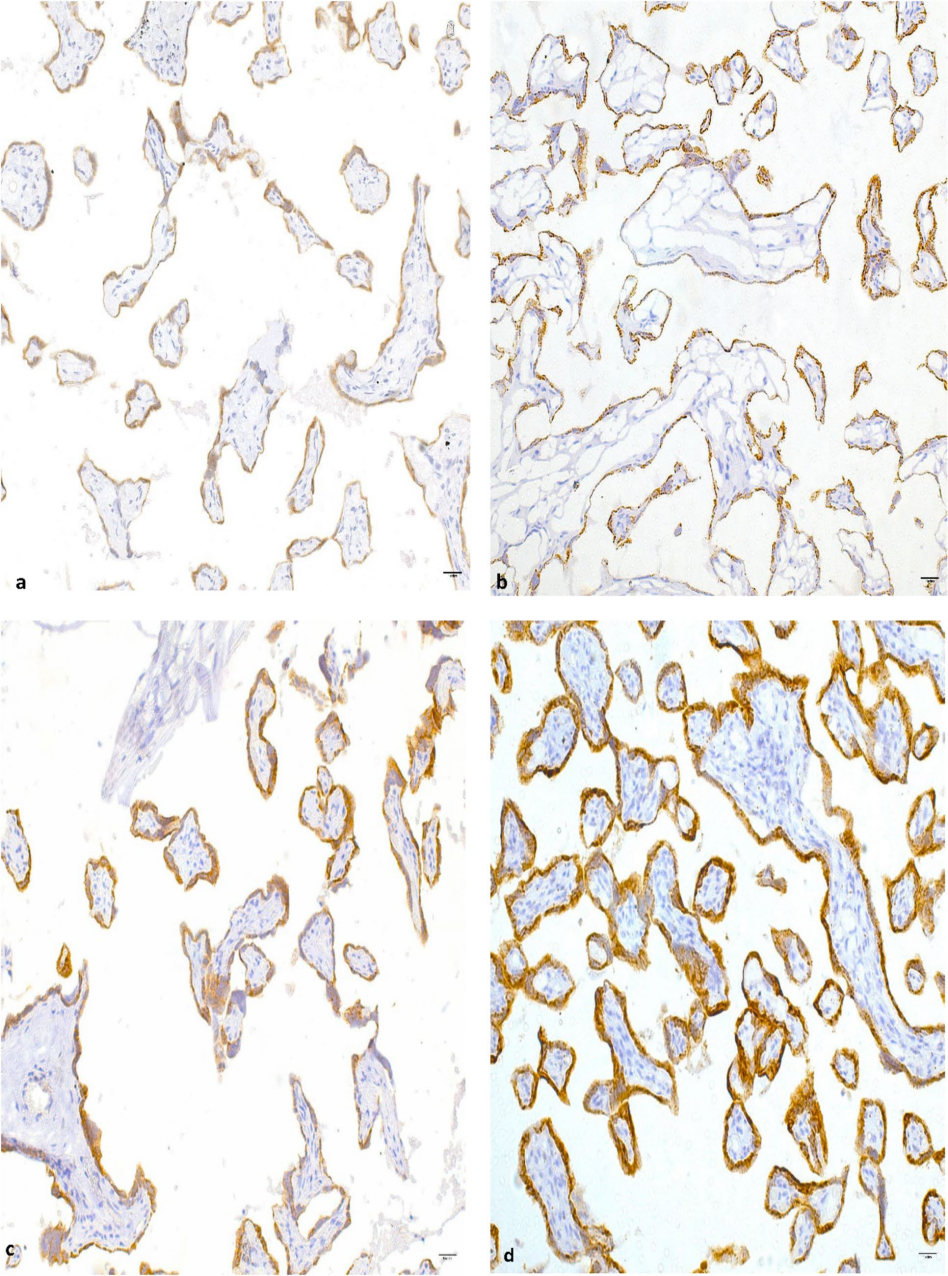
Immunohistochemical expression of kisspeptin in the: central (**a**) and peripheral (**b**) regions of the placentae from control pregnancies and central (**c**) and peripheral (**d**) regions of the placentae from COVID-19-positive pregnancies. The tile scans show kisspeptin staining (brown) of the villous syncytiotrophoblast cell layer, with nuclei (blue) that were counterstained with hematoxylin. Magnification 20×, scale bar length 100 μm

This is further depicted in Fig. 3, where kisspeptin immunostaining is localized to the villous syncytiotrophoblast cell layer of the placental villi that is indicated by DAB (brown) staining, with the nuclei counterstained blue with hematoxylin. More intense and complete kisspeptin immunostaining was observed in the syncytiotrophoblast layer from central and peripheral regions of placentae from pregnancies with COVID-19 infection in comparison with healthy pregnancies.

### Histopathological features

**Fig. 4:**
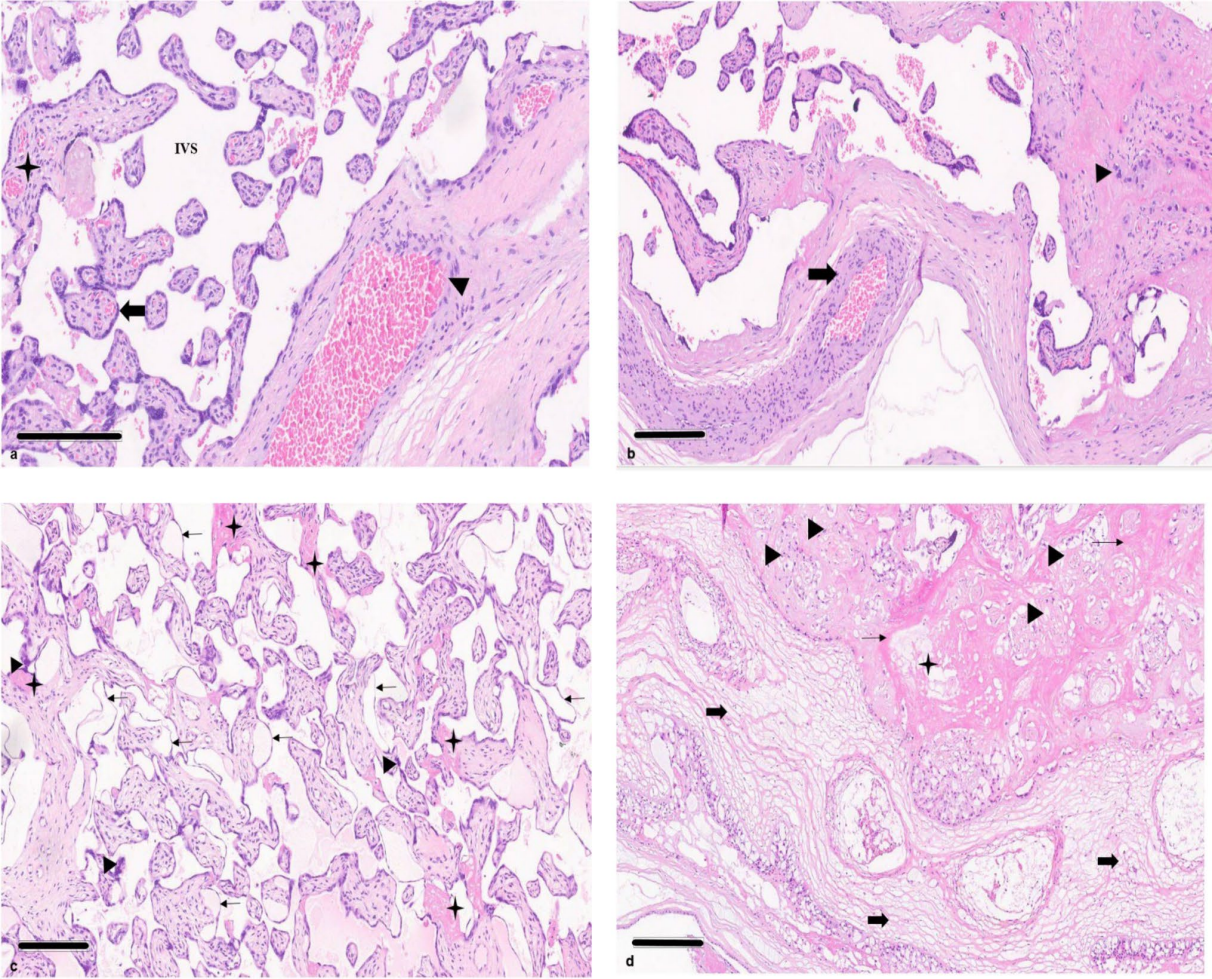
Central and peripheral placenta of control and COVID-19-positive pregnancies, stained with H&E. **a** The image shows cross-sections of the villous tree of the placenta—each chorionic villus is lined with syncytiotrophoblasts (200 µm). Inside the villi, fetal capillaries are observed. The intervillous space (IVS) is shown in white and contains maternal red blood cells. Normal well-defined intermediate villi are indicated by the arrow head. The syncytiotrophoblast layer surrounding the villi is indicated by the block arrow. Normal chorionic villi showing villous core with fetal vessels and stroma are marked by the star. **b** Normal spiral artery is indicated by the block arrow. Decidua with decidual cells characterized as epithelioid and polygonal are indicated by the arrow head (200 µm). **c** A suggestive increased fibrin deposit, along with the disappearance of nuclear material suggestive of hyalinization, are marked by the star. The figure also shows hypovascular villi characterized by villous edema and stromal fibrosis. The villi exhibit widespread trophoblast abnormalities with thinning of the villous trophoblast layer, as indicated by the line arrow. The presence of syncytial knots is indicated by the arrow head (200 µm). **d** Villous infarction, indicated by the arrowhead, is suggestive of villous necrosis, marked by the star, along with vascular wall edema, identified by the block arrow (300 µm)

**Fig. 5:**
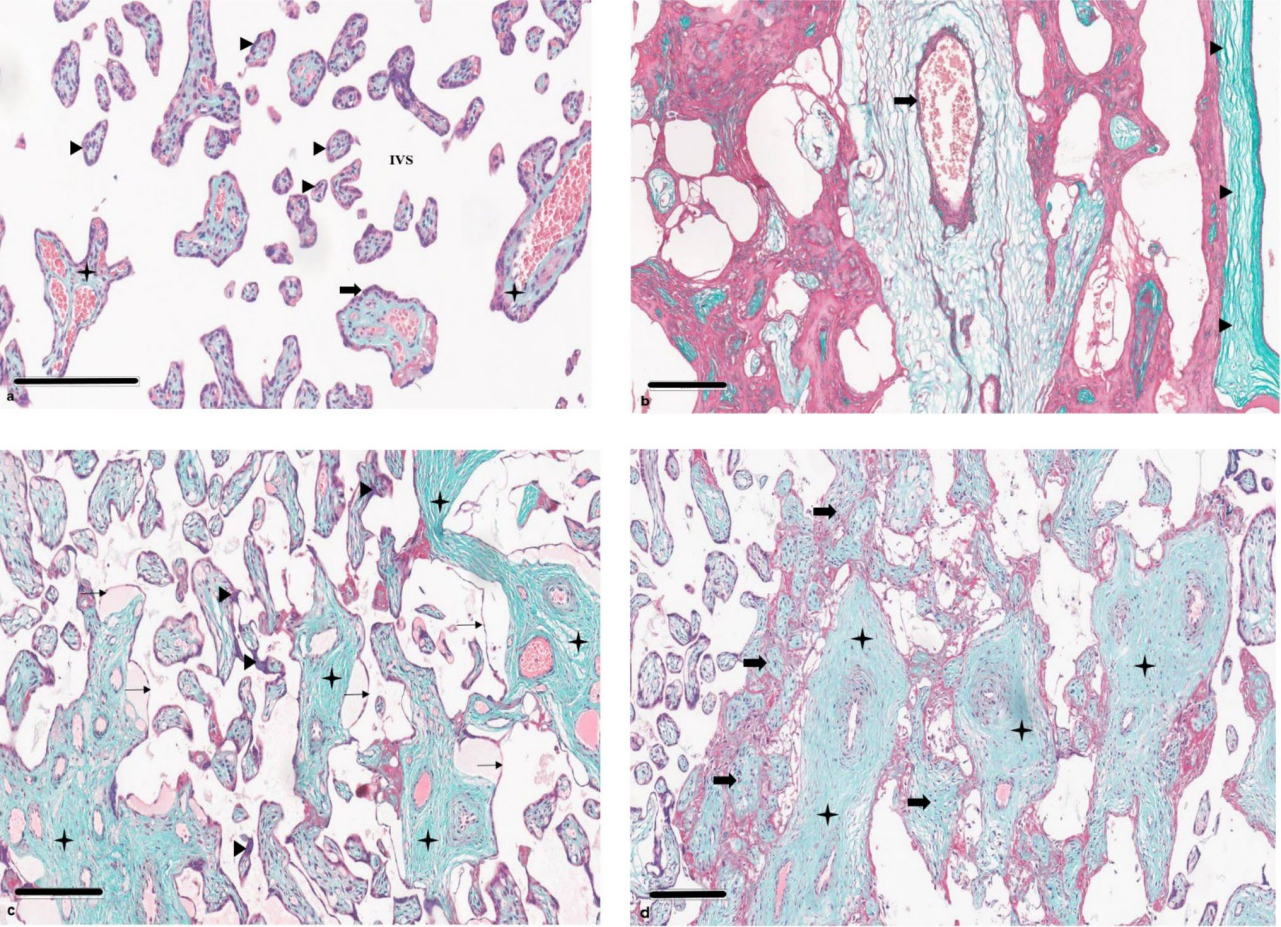
Central and peripheral placenta of control and COVID-19-positive pregnancies, stained with MT. **a** The image depicts cross-sections of the placenta’s villous tree, with each chorionic villus lined with syncytiotrophoblast cells. Inside the villi, fetal capillaries are seen. The intervillous space (IVS) is depicted in white and contains maternal red blood cells. Normal, well-defined terminal villi are indicated by the arrow head. The syncytiotrophoblast layer surrounding the villi is indicated by the block arrow. Normal chorionic villi showing a villous core with fetal vessels and stroma are indicated by the star (200 µm). **b** The normal stem artery is indicated by the block arrow. Normal chorioamniotic membranes, with no evidence of chronic inflammatory cell infiltration in the amnion and choriodecidua, are indicated by the arrow head (200 µm). **c** The increased fibrin deposit and possible hyalinization of villi are indicated by the star. The hypovascular villi with villous edema and stromal fibrosis are shown. Villi show widespread trophoblast abnormalities, with a thinning of the villous trophoblast layer, as indicated by the line arrow. An increased presence of syncytial knots are indicated with the arrow head (200 µm). **d** A massive perivillous fibrin deposition is noted with excessive deposition of fibrous tissue around the stem villi of the placenta, as indicated by the star. Hypovascular villi are marked with by block arrow (200 µm)

The histopathological findings in the control and COVID-19-positive groups are shown in Figs. 4 and 5 with a comparative evaluation in Table 2. Vascular alteration characteristics indicate maternal vascular malperfusion (MVM) or fetal vascular malperfusion (FVM), also considering inflammatory lesions. This semiqualitative evaluation (Table 2) was performed using a grading scale of 0 to 3, where 0 indicated no effect and corresponded with 0%, 1 indicated a mild effect and corresponded with 1–25%, 2 indicated a moderate effect and corresponded with 26–50%, and 3 indicated a severe effect and corresponded with values higher than 50%.

**Table 2:** Histopathological evaluation of placenta

Vascular alterations	Control						COVID-19 positive					
Histopathological features	1	2	3	4	5	6	1	2	3	4	5	6
Maternal vascular malperfusion (MVM)												
Accelerated villous maturation (increased syncytial knots)	1	1	2	2	0	1	3	3	3	2	3	3
Increased fibrin deposition	1	1	0	2	2	2	3	2	2	3	3	3
Decidual arteriopathy	1	1	2	1	3	1	3	3	3	3	3	2
Intervillous thrombosis	1	1	1	1	0	2	2	2	2	3	2	2
Retroplacental hemorrhage	0	0	0	0	1	1	0	2	2	0	0	0
Villous infarction/necrosis	0	0	0	0	0	1	0	3	3	1	2	1
Fetal vascular malperfusion (FVM)												
Avascular fibrotic villi	0	1	0	1	2	2	2	2	1	0	2	2
Distal villous hypoplasia	0	2	1	1	2	0	3	2	2	3	3	3
Chorangiosis	0	2	1	0	0	1	3	1	1	3	2	3
Delayed villous maturation (DVM)	0	0	0	0	1	1	1	2	2	1	2	2
Fetal thrombotic vasculopathy (FTV)	1	2	1	1	2	1	3	2	2	3	3	3
Intramural fibrin deposition	1	2	0	1	1	2	1	2	3	3	3	3
Vascular ectasia	1	1	0	0	1	1	3	1	2	3	3	1
Villous stromal vascular karyorrhexis	0	0	0	1	0	0	2	2	2	0	2	1
Inflammatory alterations												
Chronic villitis	0	0	0	0	0	0	2	1	1	2	2	2
Villous edema	0	1	1	1	0	0	0	3	3	3	2	2

Granding scale:

0 indicates no effect, which corresponds with 0%

1 indicates a mild effect, which corresponds with 1–25%

2 indicates a moderate effect, which corresponds with 26–50%

3 indicates a severe effect, which corresponds with > 50%

Histopathology of the COVID-19-positive placentae demonstrated a higher grade of MVM and FVM with increased villous edema and chronic villitis compared with the control placentae.

### Maternal vascular malperfusion in COVID-19-positive versus control groups

A severe effect of accelerated villous maturation was observed in five out of six COVID-19-positive placentae, with moderate effect in one out of six, as indicated by increased syncytial knots and villous agglutination. The control group showed one out of six with no effect, three out of six with mild effect, and two out of six with moderate effect. Massive perivillous fibrin deposition is the term used to describe the excessive deposition of fibrous tissue around the chorionic villi of the placenta. A severe effect of increased fibrin deposition was noted in four out of six COVID-19-positive placentae, with a moderate effect seen in two out of six in the group. In the control placentae group, moderate (*n* = 3/6), mild (*n* = 2/6), and no effect (*n* = 1/6) of increased fibrin deposition was displayed.

Decidual arteriopathy presented with thickening or fibrinoid necrosis of the vessel wall; endothelial swelling and detachment was noted. Severe effects of decidual arteriopathy were observed in five out of six COVID-19-positive placentae, with moderate effects in one out of six in the group. However, a mild effect of decidual arteriopathy was shown in four out of six, moderate effect in one out of six, and severe effect in one out of six of the control placentae.

Blood coagulation in the intervillous space can cause several pathologic lesions, the majority of which seem laminated, and are known as thrombi. A severe effect of intervillous thrombosis was observed in one out of six COVID-19-positive placenta, while a moderate effect was noted in five out of six placentae. In the control placentae group, moderate (*n* = 1/6), mild (*n* = 4/6), and no effect (*n* = 1/6) of intervillous thrombosis was observed.

When the placenta detaches over an extensive area and forms a hematoma between the uterine wall and the placenta, it produces a retroplacental hemorrhage. A moderate effect of retroplacental hemorrhage was noted in two out of six COVID-19-positive placentae, with no effect observed in four out of six of the group. Similarly, no effect (*n* = 4/6) of retroplacental hemorrhage, with a mild effect in two out of six control placentae, was exhibited. Villi that have undergone ischemic necrosis as a result of a focused reduction in placental (maternal) blood flow create placental parenchymal lesions. A severe effect of villous infarct/necrosis lesions was observed in two out of six COVID-19-positive placentae, with a further one out of six showing a moderate effect. Moreover, a mild effect in two out of six and no effect (*n* = 1/6) was also seen in the COVID-19-positive group. In contrast, a greater number of control placentae (*n* = 5/6) displayed no effects, and one out of six displayed mild effects of villous infarct/necrosis lesions.

### Fetal vascular malperfusion in COVID-19-positive versus control groups

Loss of villous vascularity with the replacement of the villus core by dense fibroblastic material is known as avascular fibrotic villi. A moderate effect in four out of six COVID-19-positive placentae of avascular fibrotic villi was observed, while mild (*n* = 1/6) and no effect (*n* = 1/6) were also noted in the group. In the control placentae group, moderate (*n* = 2/6), mild (*n* = 2/6), and no effect (*n* = 2/6) of avascular fibrotic villi was observed.

Distal villous hypoplasia was characterized by a sparse, poorly developed distal villous tree with abnormally shaped, elongated, slender villi and a widened intervillous space. The villi showed widespread trophoblast abnormalities with a thinning of the villous trophoblast layer, a reduction in cytotrophoblast numbers, and evidence of a widespread increase in syncytiotrophoblast nuclear senescence and wave-like syncytial knots. A severe effect of distal villous hypoplasia was observed in four out of six COVID-19-positive placentae, with two out of six showing moderate effect. No effect (*n* = 2/6) of distal villous hypoplasia was demonstrated in the control placentae, with two out of six showing mild and moderate effects, respectively. Capillary hyperplasia in the terminal villi is known as chorangiosis, and it results from low grade tissue hypoxia or persistent placental hypoperfusion. A severe effect of chorangiosis was demonstrated in three out of six COVID-19-positive placentae, with a further one out of six showing moderate effect. Moreover, a mild effect of chorangiosis was reported in two out of six of the COVID-19-positive group. However, no effect of chorangiosis was displayed in three out of six control placentae, with two out of six placentae showing mild effect and one out of six showing moderate effect in the group.

Delayed villous maturation (DVM) is distinguished by decreased tertiary villus production, decreased vasculosyncytial membrane formation, and, in more severe cases, enlarged bullous villi. Moderate effect of DVM was demonstrated in four out of six COVID-19-positive placentae, with mild effect noted in two out of six. In contrast, no effect of DVM was displayed in four out of six control placentae, with only two out of six placentae showing mild effect.

Fetal thrombotic vasculopathy (FTV) is a vascular thrombotic condition causing obstruction of arteries and veins in the fetal circulation of the placenta, resulting in ischemic changes in the villi peripheral to the obstruction. A severe effect of FTV was demonstrated in four out of six COVID-19-positive placentae, with moderate effect noted in two out of six. However, a moderate effect of FTV was demonstrated in two out of six control placentae, with four out of six placentae showing mild effect.

Intramural fibrin deposition occurs when abnormal amounts of fibrin is deposited in the intima of the vessel thus escalating FVM. A severe effect of intramural fibrin deposition was displayed in four out of six COVID-19-positive placentae, with moderate (*n* = 1/6) and mild effect (*n* = 1/6) noted in the group. In the control placentae group, moderate (*n* = 2/6), mild (*n* = 3/6), and no effect (*n* = 1/6) of intramural fibrin deposition were observed.

Luminal dilatation (vascular ectasia) resulting from elevated venous pressure is characterized by a pair of large fetal arteries in the chorionic plate or stem villi, one of which has a luminal diameter at least four times greater than the adjacent vessel. A severe effect of vascular ectasia was observed in three out of six COVID-19-positive placentae with moderate (*n* = 1/6) and mild (*n* = 2/6) effect also noted in the group. No effect (*n* = 2/6) of vascular ectasia was demonstrated in the control placentae, with four out of six showing mild effects.

Loss of vascular wall integrity, fragmentation and extravasation of red blood cells in the stroma, and early septation are signs of villous stromal-vascular karyorrhexis. Moderate effect of villous stromal-vascular karyorrhexis was reported in four out of six COVID-19-positive placentae, with mild effect displayed in one out of six and no effect in one out of six of the group. In contrast, no effect of villous stromal-vascular karyorrhexis was exhibited in five out of six control placentae, with only one out of six placentae showing mild effect.

### Inflammatory lesions of COVID-19-positive versus control groups

The classic chronic inflammatory placental lesion known as chronic villitis is defined by the presence of chronic inflammatory cells infiltrating the chorionic villi, which ultimately results in villous agglutination and loss of placental function. A moderate effect of chronic villitis was noted in four out of six COVID-19-positive placentae, with a mild effect seen in two out of six. In contrast, no effect of chronic villitis in any of the six control placentae was reported.

Placental villous edema was identified by locating open spaces within the cytoplasm of intervillous cells and in the interstitium of the villi. A severe effect of villous edema was reported in three out of six COVID-19-positive placentae, with moderate effect seen in two out of six and no effect in one out of six within the group. However, the control placentae group showed no effect (*n* = 3/6) and mild effect (*n* = 3/6) of villous edema.

## Discussion

The findings from this study corroborate that COVID-19 has a severe pathogenic impact on the morphology of the placenta, which may be indicative of impaired placental function. A significant elevation in the expression of kisspeptin was observed in the central and peripheral regions of the placentae from COVID-19-positive pregnancies in this study when compared with COVID-19-negative pregnancies. The placentae from these pregnancies with COVID-19 infection further presented with vascular and inflammatory alterations.

Kisspeptin has an essential role in trophoblast invasion, angiogenesis, and placentation; therefore, any alterations in its expression could result in adverse pregnancy outcomes or placental dysfunction (Kapustin et al. 2020). This is due to kisspeptin inhibiting the matrix metalloproteinase-9 (MMP9) expression in the placenta, which negatively impacts the maturation and invasion; thus, the elevated expression of kisspeptin can consequently result in gestational complications (Zhang et al. 2011; Hu et al. 2019; Kleimenova et al. 2019). Elevations in placental kisspeptin have therefore been linked to inadequate trophoblast invasion, which is inhibited by kisspeptin (Cartwright and Williams 2012; Matjila et al. 2016). Hence, it is evident from the results reported herein that COVID-19 has a significant impact on placental functioning via the alteration of the placental kisspeptin expression, which could possibly impact sufficient trophoblast invasion in these pregnancies. To our knowledge, these results are yet to be supported by other studies. However, studies on preeclampsia, gestational diabetes mellitus, and preterm birth have noted similar elevations in the placental expression of kisspeptin (Torricelli et al. 2008; Zhang et al. 2011; Qiao et al. 2012; Vodneva et al. 2014; Matjila et al. 2016; Kapustin et al. 2020). We believe this alteration in kisspeptin could be linked to the inflammation observed in COVID-19 pregnancies. Furthermore, the cytokine profile was noted to be altered in the plasma and extracellular vesicles of the current cohort, in which we observed a proinflammatory state (Heeralall et al. 2025) . The signaling of KISS-1 systems are sensitive to inflammatory conditions; therefore, this environment could disrupt its signaling, accounting for the altered kisspeptin expression observed in our study (Iwasa et al. 2008). We believe that the placental dysfunction observed in COVID-19 pregnancies could be attributed to the kisspeptin alterations observed in this study and could have further manifested as morphological alterations.

The alteration in this key protein was further supported by the placental histopathological changes observed in the placentae from COVID-19-positive pregnancies in the South African cohort. Signs of morphological alterations indicative of maternal and fetal vascular malperfusion were evident in all of the COVID-19-positive placentae when compared with placentae from COVID-19-negative pregnancies. This was identified in the South African cohort through the increased presence of pathological changes, including increased fibrin deposition, intervillous thrombosis, necrosis, chorangiosis, and fetal thrombotic vasculopathy, when compared with the control group. Other South African studies also noted alterations in most of the COVID-19 positive placentae analyzed in their studies (Vannevel et al. 2021; Nunes et al. 2022; Ramphal et al. 2022). Similar observations have been noted in other countries, including Switzerland, Italy, USA, India, Netherlands, Belgium, Brazil, France, China, Korea, and Uzbekistan, in which an increased prevalence of MVM and FVM in COVID-19 placentae was also identified when compared with controls (Baergen and Heller 2020; Baud et al. 2020; Facchetti et al. 2020; Hecht et al. 2020; Mongula et al. 2020; Mulvey et al. 2020; Pulinx et al. 2020; Richtmann et al. 2020; Shanes et al. 2020; Sisman et al. 2020; Smithgall et al. 2020; Vivanti et al. 2020; Gao et al. 2021; Ikhtiyarova et al. 2021; Jaiswal et al. 2021; Jang et al. 2021; Menter et al. 2021; Patberg et al. 2021; Watkins et al. 2021; Dubucs et al. 2022; Londhe et al. 2024; Tripathy et al. 2024). In addition to the increased prevalence of malperfusion, a significant increase in inflammation was observed in all the COVID-19 placentae that presented with severe chronic villitis and villous edema compared with the placentae from the control group. This could possibly be a result of the altered proinflammatory cytokine profile noted in the South African cohort from this study (unpublished data). Similar findings were recorded worldwide in the Italian, American, Brazilian, Chinese, Korean, Swiss, French, Indian, Indonesian, and Turkish populations, confirming that COVID-19 results in inflammatory and vascular alterations (Facchetti et al. 2020; Hecht et al. 2020; Richtmann et al. 2020; Gao et al. 2021; Jang et al. 2021; Menter et al. 2021; Dubucs et al. 2022; Huynh et al. 2022; Garg et al. 2023; Milot et al. 2023; Altuntaş et al. 2024; Ryan et al. 2024; Umamaheswari et al. 2024; Wardhana et al. 2024).

The altered placental kisspeptin expression and increased vascular and inflammatory alterations observed in this study can be further linked to the increase in distress and death in neonates from mothers infected with COVID-19. This study notes that three of six (50%) neonates from COVID-19-positive mothers experienced distress compared with only one of six (16.67%) neonates from COVID-19-negative mothers. Furthermore, three neonates from COVID-19-positive pregnancies were born premature or presented with abnormalities and died, while no abnormalities or deaths were noted in neonates from COVID-19-negative pregnancies. Similarly, an increased prevalence of alterations was reported in the COVID-19 positive placentae from human immunodeficiency virus (HIV)-positive patients when compared with HIV-negative patients in the South African cohort, which was further reported to be associated with preterm delivery (Barbera et al. 2021; Nunes et al. 2022; Ramphal et al. 2022). Furthermore, this population could have been more susceptible to COVID-19 complications owing to the increased prevalence of social and healthcare issues that could have exacerbated the effects of COVID-19 (Lone and Ahmad 2020).

Therefore, from the results presented herein, we believe that the altered cytokine profile observed in this population impacted kisspeptin signaling that was demonstrated through increased placental kisspeptin expression in COVID-19-positive pregnancies. This alteration could have impacted trophoblast invasion, suggesting that spiral artery conversion is impacted along with blood flow that could lead to poor placental perfusion and hypoxia. These conditions, along with hyperinflammation, could be the reason for the placental dysfunction observed through the altered histopathology in the COVID-19 placentae. However, the neonates from these pregnancies remain a concern as it is clear that they were exposed to environments that were not conducive to optimal development and growth. Studies have already reported complications that impact fetal cardiac function and morphology in infants from pregnancies affected by COVID-19 (Zhu et al. 2024).

## Conclusions

This study shows that COVID-19 infection during pregnancy impacts placental functioning owing to the potential influence of altered placental kisspeptin expression. This alteration could be linked to the vascular and inflammatory morphological alterations observed in the COVID-19-positive placentae in this study. This study suggests a plausible association between elevated placental kisspeptin expression and histopathological alterations.

## Limitations

During the final wave of the COVID-19 pandemic, the collecting site still followed strict regulations, and access to patients and their clinical data was restricted. Therefore, this contributed to a limited sample size and lack of vaccination status. Nevertheless, our findings provide valuable insights into the critical role that kisspeptin plays in COVID-19 pregnancies and highlights the importance of conducting more research on COVID-19 pregnancies.

## Future recommendations

The long-term effects of COVID-19 in pregnancies remain poorly elucidated, necessitating further investigation into potential impacts. Investigating placentation in COVID-19 pregnancies is essential in understanding pathology, identifying adverse outcomes, and exploring potential therapeutic interventions in managing these complications. In particular, more focused research should be conducted on kisspeptin in COVID-19 positive pregnancies. Additionally, it is vital for the mothers and neonates from these pregnancies to be rigorously monitored so that any detrimental outcomes as a result of COVID-19 can be identified and treated swiftly, minimizing long-term consequences in these patients.

## Data Availability

No datasets were generated or analyzed during the current study.
